# Effects of preoperative serum lactate dehydrogenase levels on long-term prognosis in elderly patients with hepatocellular carcinoma undergoing transcatheter arterial chemoembolization

**DOI:** 10.3389/fsurg.2022.982114

**Published:** 2022-09-23

**Authors:** Yuantao Gan, Fengwei Gao, Bo Du, Yu Liu, Qian Xue, Jinqiang Fu

**Affiliations:** Department of Hepatopancreatobiliary Surgery, Leshan People’s Hospital, Leshan, China

**Keywords:** transcatheter arterial chemoembolization, elderly, hepatocellular carcinoma, lactate dehydrogenase, long-term prognosis

## Abstract

Hepatic arterial chemoembolization is an effective treatment for primary hepatocellular carcinoma (HCC) and can improve the survival rate of patients. Nevertheless, the long-term prognosis of patients with HCC is not optimistic. In recent years, tumor humoral detection has attracted extensive attention and is expected to become the main examination method for early tumor screening. Studies have found that serum LDH is an indicator with effective potential to predict tumor proliferation and progression, such as pancreatic cancer, esophageal cancer, nasopharyngeal cancer, etc., but the relationship between this indicator and the prognosis of HCC is still unclear. The purpose of this study was to clarify the relationship between serum LDH and the prognosis of patients with HCC, so as to provide an important scientific basis for prognosis judgment of HCC.

## Introduction

Hepatocellular carcinoma (HCC) is abbreviated as liver cancer. As a common malignant tumor, it has a high prevalence in middle-aged men. Although the current treatment technology has been continuously improved, there are still studies reporting that HCC patients recur within 5 years. The rate is as high as 60% or more (1, 2). At present, surgical resection is the preferred method for the treatment of HCC. However, due to the high incidence of postoperative adverse reactions and many surgical contraindications, its clinical application is limited (3, 4). Therefore, the treatment of HCC patients still needs to adopt the form of comprehensive treatment of multiple methods. Among them, an important non-surgical method for the treatment of HCC patients is hepatic arterial chemoembolization (5). Lactate dehydrogenase (LDH) is a key metabolic enzyme in glycolysis, which can reflect the liver function *in vivo* (6, 7). However, the research on serum LDH level to help diagnose cancer or judge the prognosis is still in the primary stage, especially the correlation research on predicting the prognosis of HCC is few. In this study, 106 elderly patients with HCC who underwent hepatic arterial chemoembolization in our hospital were investigated, and the effect of preoperative serum LDH levels on long-term prognosis was analyzed. The purpose of this study was to explore the value of LDH level in evaluating the long-term prognosis of HCC patients, and then to provide certain schemes and strategies for clinical treatment.

## Materials and methods

### General information

The random number table method was used to randomly select 106 elderly HCC patients who underwent hepatic arterial chemoembolization in our hospital from January 2011 to December 2013. Among them, 57 patients with preoperative serum LDH level ≤400 U/L were selected as the treatment group, and 49 cases with LDH level higher than 400 U/L were the control group. The content of this study has been reviewed and approved by the Medical Ethics Committee of our hospital, and 106 patients and their families voluntarily signed an informed notice.

### Inclusion criteria

According to the imaging examination results, clinical symptoms, pathological tissue biopsy and clinical biochemical treatment comprehensive evaluation, the diagnosis of HCC was made; Patients diagnosed for the first time; The patient's bile duct vessels were not invaded by the tumor; no tumor recurrence was found in the review of tumor markers and imaging 3 months after treatment.

### Exclusion criteria

HCC metastasis; Associated with other malignancies; With spontaneous rupture bleeding; with hepatitis C, syphilis, AIDS, etc.; with mental illness.

### Treatment methods

In addition to conventional treatments such as anti-tumor and liver protection, both groups were treated with hepatic arterial chemoembolization, and the Seldinger method was used for percutaneous arterial puncture. The catheter was selectively inserted into the blood supply artery of hepatocytes, and angiography was performed to determine the distribution of tumor blood vessels, the blood supply artery of liver tumor and the area of tumor foci. 15 ml of mixed injection of THP, carboplatin, lipiodol emulsion and 5-fluorouracil was perfused into the catheter, and the amount of 1–2 ml could be appropriately increased according to the actual situation of the patient. After completion, the catheter was taken out, and the puncture site was pressed to stop the bleeding, and absolute bed rest ≥2 days. Repeat the treatment every other month for 3–4 times in total, in order to block the blood supply of cancer cells, and then fight against tumor cells.

### Detection of serum LDH levels

The same automatic biochemical analyzer (produced by Beckman Coulter, USA, model AU680) was used to analyze the preoperative 7 days and postoperative 1 day, 7 days, 1 month, 6 months, 12 months and 3 years of the two groups. The serum LDH levels of the two groups were detected, and the detection operations of the two groups of patients were carried out by the same laboratory physician in our hospital strictly according to the instructions.

### Efficacy evaluation criteria

The clinical efficacy evaluation is divided into markedly effective, effective, general and invalid. (1) markedly effective: the reduction of the tumor focus is 5 cm or more, the alpha-fetoprotein is less than 20 mg/L, and the proliferation activity of the cancer cells disappears completely; (2) Effective: the reduction range of cancer is 3–5 cm, the level of alpha-fetoprotein is 20–150 mg/L, and the proliferation activity of cancer cells disappears; (3) General: The cancer shrinkage range is 1–3 cm, the alpha-fetoprotein level is 150–400 mg/L, and the cancer cells still have proliferation activity; (4) Invalid: Cancer shrinks less than 1 cm, alpha-fetoprotein level is higher than 400 mg/L, and cancer cells are actively proliferating (8).

### Statistical methods

The clinically relevant data of the two groups of patients were entered into SPSS 21.0 statistical software for data processing and analysis. The measurement data such as LDH levels at each time point were expressed by ($\bar{x}\pm \text{s}$) and t-test was used, while the enumeration data such as the markedly effective rate was used, and the effective rate were expressed as percentages (%), then the *χ*^2^ test was used, and *P *< 0.05 was considered to be statistically significant.

## Results

### Comparison of general clinical data of the two groups of patients

There were no significant differences in general data such as sex ratio, age, tumor diameter, tumor number, liver function grading, cytological type, histological differentiation degree and clinical stage between the two groups of patients (*P *> 0.05), which was comparable. As shown in Table 1.

**Table 1:** Comparison of general clinical data of the two groups of patients.

Group	Sex ratio (male:female)	Age (year)	Tumor diameter (cm)	The number of tumors (pieces)	Liver function classification (*n*)		
					Child-Pugh A stage	Child-Pugh B stage
Control group (*n* = 49)		31:18	54.75 ± 4.92	2.87 ± 0.86	1.79 ± 0.66	29	20	
Test group(*n* = 57)		35:22	53.45 ± 4.76	2.76 ± 0.84	1.64 ± 0.75	32	25	
*χ*^2^/*t*	0.96	−0.79	−0.35	−0.47	0.67			
*P*	0.21	0.25	0.62	0.58	0.43			
Group	Cytological typing (*n*)		Degree of histological differentiation (*n*)			Clinical stage (*n*)		
	Hepatocellular carcinoma	Cholangiocarcinoma	Poorly differentiated	Moderate differentiation	Highly differentiated	Phase I	Phase II	Phase III
Control group (*n* = 49)	40	9	9	39	1	17	31	1
Test group (*n* = 57)	45	12	11	42	4	21	33	3
*χ* ^2^	0.74		0.98			0.79		
*P*	0.38		0.20			0.37		

### Serum LDH levels of patients in the two groups at various time points after operation

One year after operation, 1 patient in the treatment group was lost to follow-up, and all patients in the control group were followed up. Three years after surgery, 3 patients in the treatment group were lost to follow-up, and 2 patients in the control group were lost to follow-up. One day after operation, there was no significant difference in serum LDH levels between the two groups (*P *> 0.05). At 7 days, 1 month, 6 months, and 12 months after operation, the levels of serum LDH in the two groups were lower than those at 1 day after operation (*P *< 0.05), and the levels in the treatment group were significantly lower than those in the control group (*P *< 0.05). At 3 years after operation, serum LDH levels in both groups were lower than at 1 day after operation (all *P *< 0.05), but there was no statistical significance between the two groups (*P* > 0.05). As shown in Table 2.

**Table 2:** Serum LDH levels at each time point after surgery in the two groups of patients ($\bar{x}\pm \text{s}$, U/L).

Group	*n*	1 day after surgery	7 d after surgery	1 month after surgery	6 month after surgery	12 month after surgery	3 years after surgery
Control group	47	409.75 ± 28.54	389.75 ± 27.46	333.86 ± 29.54	306.86 ± 24.75	292.75 ± 24.86	257.35 ± 21.54
Test group	54	397.46 ± 23.75	342.85 ± 23.75	299.75 ± 23.75	268.57 ± 23.75	257.86 ± 19.54	244.65 ± 19.43
*t*		−1.04	−4.86	−4.57	−4.68	−4.35	−2.89
*P*		0.12	<0.01	<0.01	<0.01	<0.01	0.03

### Comparison of curative effect of two groups of patients 1 year after operation

The curative effect rate (39.29%) of the treatment group was higher than that of the control group (22.92%) at 1 year after operation (*P* < 0.05), and the general curative effect rate (17.86%) and inefficiency (12.50%) were lower than those of the control group (27.08%)., 20.83%) (both *P *< 0.05). As shown in Table 3.

**Table 3:** Comparison of curative effect of two groups of patients at 1 year after operation [*n*(%)].

Group	*n*	Significant effect	Valid	General effect	Invalid
Control group	48	11 (22.92)	14 (29.17)	13 (27.08)	10 (20.83)
Test group	56	22 (39.29)	17 (30.36)	10 (17.86)	7 (12.50)
*χ* ^2^		5.56	0.32	4.87	4.37
*P*		0.02	0.62	0.03	0.04

### Comparison of curative effect of serum LDH decreased or increased 3 years after operation

The LDH levels measured at 3 years after surgery were compared with the levels at 1 year after surgery, and it was found that serum LDH levels decreased in 70 patients, while serum LDH levels increased in 41 patients. 3 years after operation, the markedly effective rate (37.14%) and effective rate (41.43%) of patients with decreased serum LDH level were significantly higher than those with increased serum LDH level (19.51%, 29.27%) (*P *< 0.05), the inefficiency (4.29%) was significantly lower than that of those with increased serum LDH level (29.27%) (*P *< 0.05). As shown in Table 4.

**Table 4:** Comparison of curative effects of patients with decreased or increased serum LDH 3 years after operation

Group	*n*	Significant effect	Valid	General effect	Invalid
LDH reduce	70	26 (37.14)	29 (41.43)	12 (17.14)	3 (4.29)
LDH raise	41	8 (19.51)	12 (29.27)	9 (21.95)	12 (29.27)
*χ* ^2^		7.94	4.92	0.94	12.43
*P*		<0.01	0.03	0.21	<0.01

## Discussions

HCC is one of the third largest tumors of the digestive system, with a high degree of malignancy and rapid disease progression, and its prevalence has been increasing year by year in the past decade (9, 10). The liver plays an important compensatory role in the human body, and at the same time, it usually undergoes changes from hepatitis to cirrhosis before the occurrence of HCC. However, it is difficult to effectively detect early cancer by clinical biochemical indicators. therefore, when liver failure occurs and the corresponding symptoms, most patients have entered the advanced stage of liver cancer. at this time, the best opportunity for treatment has often been lost and the life safety of patients has also received a serious threat (11, 12). After the liver becomes cancerous, cancer cells can spread rapidly in a short period of time, and patients with advanced liver cancer often develop severe jaundice, liver tumors and systemic symptoms (13). Especially in elderly patients, due to their low immune function and poor tolerance, the degree of body failure is relatively severe. At the same time, elderly patients are often complicated by various underlying diseases such as cardiovascular and cerebrovascular diseases, which ultimately lead to higher mortality (14). Transcatheter arterial chemoembolization is an important non-surgical method for the treatment of patients with liver cancer, and its clinical efficacy is ideal, which can effectively prolong the survival time of patients (15). At the same time, it can assist radical resection of liver cancer to play a synergistic role, effectively remove residual cancer cells, thereby effectively enhancing the short-term and long-term efficacy of patients, helping to reduce the disease recurrence rate and improve the prognosis of patients (16).

In this study, we found that the serum LDH levels of patients in the two groups were lower than those in the control group 1 day, 1 month, 6 months, and 12 months after surgery. The results suggested that LDH levels of patients with low preoperative LDH level or high preoperative LDH level could be reduced to a certain extent after hepatic artery chemoembolization, and the reduction amplitude of LDH concentration after operation in patients with low preoperative LDH level was significantly greater than that in patients with high preoperative LDH level. As one of the important enzymes in the process of glycolysis, LDH can exist in the cytoplasm of all tissues and cells of the body, especially in the liver (17). Because the distribution of LDH isozyme has good tissue specificity, it can be used for clinical diagnosis according to its tissue specificity (18). In HCC, because the intensity and speed of metabolism and necrosis of cancer cells are higher than those of normal cells, their cell membrane permeability is prone to change, resulting in the release of more enzymes in cancer cells into serum, which in turn leads to an increase in serum LDH concentration. Studies have reported that the concentration of serum LDH in HCC patients is about 35% higher than that in normal liver, and the positive detection rate of serum LDH for HCC is about 78% (19). It is considered that the detection of serum LDH level is one of the convenient and feasible methods. At the same time, it can not only reflect the metabolism and proliferation of cells, but also reflect the state of glycolysis, anaerobic or malignant lesions when the liver cells become cancerous in the early stage. In addition, changes in LDH serum levels directly or indirectly reflect the strength of glycolysis, thereby predicting the tumor proliferation and development ability. Therefore, some studies (20) have pointed out that serum LDH is one of the important indicators for evaluating the short-term and long-term efficacy of interventional therapy in patients with HCC.

This study found that the curative effect rate in the treatment group was higher than that in the control group at 1 year after surgery, and the general curative effect rate and inefficiency rate were lower than those in the control group. The results suggest that the high concentration of serum LDH in HCC patients will have a certain impact on the prognosis after interventional therapy. At the same time, this study found that 3 years after surgery, the markedly and effective rates of patients with decreased serum LDH levels were significantly higher than those with increased serum LDH levels, and the inefficiency was significantly lower than those with increased serum LDH levels. The results showed that compared with those with high levels of serum LDH, those with low levels were more helpful to improve the long-term prognosis of elderly HCC patients undergoing hepatic arterial chemoembolization, thereby enhancing their clinical efficacy and prolonging survival time. The reason is that high LDH level may promote tumor occurrence and development by changing the *in vivo* environment and metabolism (21). Therefore, people with high LDH level are more prone to local infiltration, lymph node metastasis, and accelerated cancer progression, affecting the long-term prognosis of elderly HCC.

In conclusion, the detection of preoperative serum LDH level is helpful to evaluate the long-term prognosis of elderly HCC patients undergoing hepatic arterial chemoembolization. The low level of LDH in serum can also reflect the long-term curative effect of the patient, which can provide a certain strategy for selecting a more appropriate and effective program for clinical treatment. However, this study has the following shortcomings: (1) The small sample size included may lead to statistical differences, and it is necessary to further increase the sample size and improve the statistical strength in the future; (2) This study is a retrospective single-center study. There may be some uncontrollable factors or interfering factors in the baseline data of included subjects, and multi-center and prospective studies shall be designed for further verification in the future.

## Data Availability

The original contributions presented in the study are included in the article/Supplementary Material, further inquiries can be directed to the corresponding author/s.
